# Cineradiographic Analysis of Mouse Postural Response to Alteration of Gravity and Jerk (Gravity Deceleration Rate)

**DOI:** 10.3390/life4020174

**Published:** 2014-04-24

**Authors:** Katsuya Hasegawa, Priscila S. de Campos, Jorge L. Zeredo, Yasuhiro Kumei

**Affiliations:** 1 JAXA/Institute of Space and Astronautical Science, Sagamihara 252-5210, Japan; E-Mail: hasegawa@keisoku.jaxa.jp; 2 Graduate Program in Health Sciences and Technology, University of Brasilia, Brasilia 72220-140, DF, Brazil; E-Mails: psales.campos@hotmail.com (P.S.C.); jllzeredo@unb.br (J.L.Z.); 3 Department of Hard Tissue Engineering, Tokyo Medical and Dental University Graduate School, Tokyo 113-8549, Japan

**Keywords:** gravity, jerk, parabolic flight, threshold, spine, partial gravity, reflex, space, kinesiology, cineradiography

## Abstract

The ability to maintain the body relative to the external environment is important for adaptation to altered gravity. However, the physiological limits for adaptation or the disruption of body orientation are not known. In this study, we analyzed postural changes in mice upon exposure to various low gravities. Male C57BL6/J mice (*n* = 6) were exposed to various gravity-deceleration conditions by customized parabolic flight-maneuvers targeting the partial-gravity levels of 0.60, 0.30, 0.15 and μ *g* (<0.001 *g*). Video recordings of postural responses were analyzed frame-by-frame by high-definition cineradiography and with exact instantaneous values of gravity and jerk. As a result, the coordinated extension of the neck, spine and hindlimbs was observed during the initial phase of gravity deceleration. Joint angles widened to 120%–200% of the reference *g* level, and the magnitude of the thoracic-curvature stretching was correlated with gravity and jerk, *i.e.*, the gravity deceleration rate. A certain range of jerk facilitated mouse skeletal stretching efficiently, and a jerk of −0.3~−0.4 *j* (*g*/s) induced the maximum extension of the thoracic-curvature. The postural response of animals to low gravity may undergo differential regulation by gravity and jerk.

## 1. Introduction

Within their particular physiological limits, terrestrial life forms have the ability to adapt to changes in the physical properties of the environment, such as temperature, atmospheric pressure, humidity and other conditions. A certain degree of adaptability to changes in gravity is expected, but the physiological limits to these changes remain unclear [1,2,3,4]. The distressing symptoms of exposure to microgravity (μ *g*) have been reported extensively. Various effects of microgravity in humans include the upward shift in the distribution of bodily fluids [5,6], reduced sensory perception [7,8,9] and abnormalities in motor function [7,10,11,12]. However, markedly less is known about the adaptability to less severe gravitational conditions. The low-gravity levels between 1 *g* (the unit of gravity acceleration on the Earth) and μ *g* (<0.001 *g*) have been termed “partial gravity” [13]. Recent studies have attempted to examine the physiological changes that occur in partial-gravity conditions, such as those encountered on the moon (≈0.16 *g*) or on Mars (≈0.38 *g*) [14,15,16,17,18,19,20]. 

An important aspect of environmental adaptation is the establishment of orientation [21]. Mismatch between sensory modalities is believed to cause disorientation and motion sickness in humans and rodents exposed to microgravity [22,23]. However, the precise mechanism underlying the rodent mode of response to low gravity is unknown. Recently, we clarified for the first time that rats show a set of behaviors upon exposure to different levels of partial gravity, including extended body postures at <0.15 *g* [17]. The purpose of the present study was to examine the rodent response to gravity deceleration, *i.e.*, the period of transition from normal gravity to partial gravity. For this purpose, a parabolic flight maneuver was customized in the present study to create various conditions of gravity deceleration by targeting specific partial-gravity levels. A detailed analysis of postural changes was made possible by the use of a cineradiographic technology and by the moment-to-moment analyses of the instantaneous values of gravity and jerk, *i.e.*, the gravity deceleration rate.

## 2. Experimental Section

The methods described here follow ethical guidelines and received approval by the Animal Welfare Committees of Tokyo Medical and Dental University (approval No. 0120330A) and JAXA, *i.e.*, the Japan Space Agency (approval No. 012-002) to Yasuhiro Kumei. 

### 2.1. Animals

Due to the limitation of cineradiographic technology on board, a smaller rodent, mouse, was used in the present study. Young male C57BL/6J mice (age: 7 weeks, bw: 23–25 g, *n* = 6) were acquired from a breeding company (Charles River Laboratories Inc., Japan) and delivered to a laboratory at Nagoya Airport, where the animals were habituated for five days before the flight experiments. The animals were housed in pairs and kept in a temperature-controlled room (23 ± 1 °C) under a 12 h:12 h light/dark cycle (lights on from 7:00 to 19:00). All mice were allowed *ad libitum* access to water and standard chow while in the colony room.

### 2.2. Parabolic-Flight Protocol

**Figure 1:**
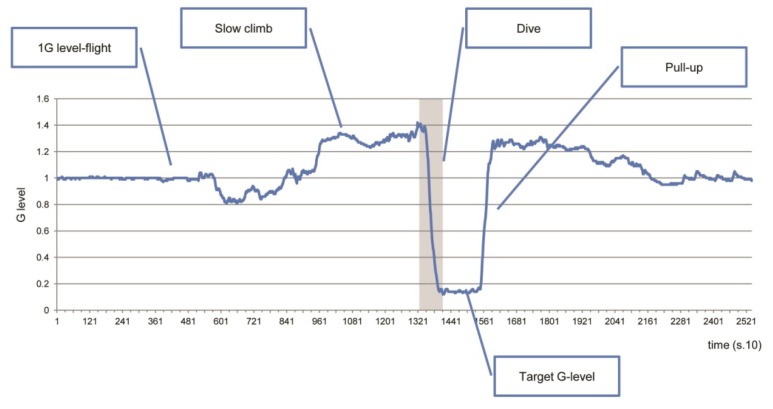
Typical parabolic flight profile. Shaded area represents the “dive” phase of the parabolic flight, where the aircraft loses altitude rapidly with gravity deceleration towards a target *g* level (in this example, the target gravity was 0.15 *g*).

Parabolic flights were operated by Diamond Air Service, Inc. (Aichi, Japan) on a Mitsubishi MU300 jet aircraft. Two flights were scheduled for this experiment on two consecutive days. Each flight had a total duration of approximately 2 h, including take-off, transit to the test area, parabolic maneuvers, transit back to the airport and landing. Our original protocol consisted of 12 parabolas, each of which aimed a specific target gravity level. On both flights, 12 parabolas were conducted in three cycles of four low-gravity levels targeting 0.6, 0.3, 0.15 and μ *g*, in that order. The cycles were preceded and followed by the 1 *g* level flight of 10 min in duration. In the present study, the flight maneuver for each parabola consisted of five phases: the 1 *g* level flight, then, a “slow-climb” phase of approximately 30 s at 1.3 *g* hypergravity, followed by a “dive” phase of gravity-change lasting 3–4 s, then a constant phase at the “target *g* level” for 10–15 s and, finally, a “pull-up” phase of approximately 30 s at 1.3 *g* for recovery to the 1 *g* level flight (Figure 1). Each parabola was separated by a 5 min interval of the 1 *g* level flight. 

### 2.3. Cineradiographic Imaging

**Figure 2:**
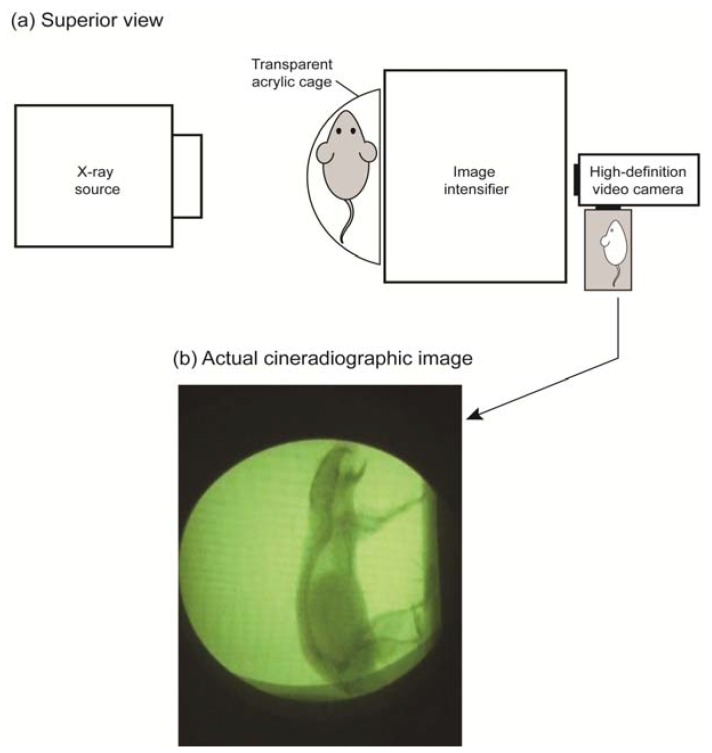
Schematic drawing of the cineradiography apparatus. (**a**) The superior view shows the test cage (semi-circular footprint) holding a mouse between the X-ray source and the image intensifier. X-ray images were captured at 25 Hz by a high-definition video camera. (**b**) The cineradiographic image shows a still-frame from the X-ray movie. The extension of the jaw, neck, spine and limbs was observed in response to the instantaneous gravitational condition of 0.153 *g* and −0.180 *j* (jerk, *g/*s).

On each flight, three mice were loaded onto the aircraft. Neither food nor water was supplied to the mice during the flight. The temperature inside the cabin was kept at 20–23 °C. One mouse was loaded into the cineradiographic apparatus (Figure 2) during each cycle of parabolas (details are above). The X-ray video system was custom-made for our flight experiments, where X-rays were emitted perpendicular to the animal at 70 kV and 0.3 mA by an X-ray tube (Toshiba Electron Tubes and Devices Co., Ltd., Ohtawara, Japan). The X-ray photons passed through the mouse and reached the 100-mm diameter entrance field of the high-speed response-type beryllium image intensifier (E5889BP-P1K, Toshiba Electron Tubes and Devices Co., Ltd., Ohtawara, Japan). Within the image intensifier, a scintillator converted the X-ray photons to light photons, which are then converted to photoelectrons within the photocathode. These electrons were accelerated and focused by electrodes, finally striking the output phosphorus screen, which converted the accelerated electrons back into visible-light photons, creating a smaller and brighter image that was captured at 25 Hz by a digital video camera on the output window. The images produced by X-rays were the result of the different absorption rates of different tissues. Bones absorb X-rays the most and look black on the radiograph, while soft tissues absorb less and look gray. 

### 2.4. Data Collection

The gravity-profiles inside the aircraft’s cabin were obtained throughout the flight by an accelerometer at the sampling rate of 10 Hz. Data of the gravity were collected for the x-, y-, z-axes. The gravitational force was synthesized from gravity measurements in the x-, y-, z-axes. The gravity and the jerk were obtained by the following formula. The optimized jerk was obtained as the gravity acceleration rate by averaging a total of 10 instantaneous values in the range of 0.5 s before and after the exact moment for the X-ray photo frame. The unit of jerk (*j*) is defined as *g*/s.

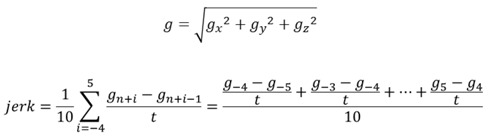

n: time (sec × 10) in gravity-data acquisition at 10Hz. t = 0.1.
g_n_: gravity-data at time “n” when the photo frame was obtained.

**Figure 3:**
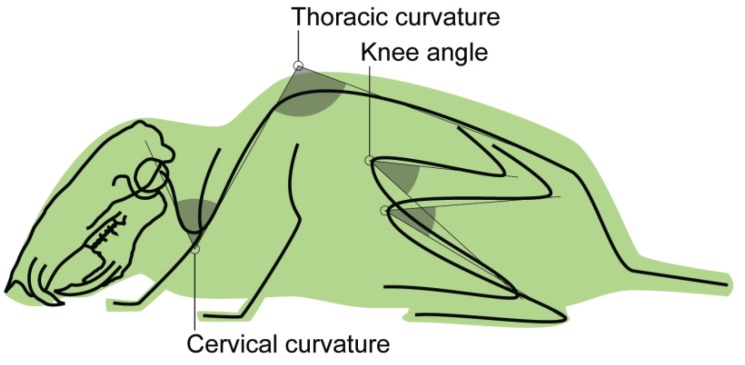
Schematic view of the mouse body showing the angles taken for postural analysis. See Table 1 for detailed explanations.

A close examination of X-ray videos taken from mice during the parabolic flight showed that postural adjustments took place at the first few seconds of the parabola, when the gravity acceleration was changing. X-ray videos were analyzed frame-by-frame, and the mice responses to gravity manipulation were measured. For data collection, frames at one second prior to the starting point of the “dive” phase were selected as the control of the pre-stimulating condition, where the animals were sitting quietly at rest in the test cage. Then, the frames from 1.0, 2.0, 3.0 and 4.0 s in the “dive” phase of the parabola, where the animals displayed greater postural change while still maintaining a foothold on the cage floor, were taken. Several landmarks were identified on the mouse skeleton from which angular measurements were made (Figure 3 and Table 1). 

**Table 1:** List of angular measurements.

Type	Label	Measurement
angle	cervical curvature	angle between cervical and thoracic segments of the spinal column
angle	thoracic curvature	angle between thoracic and lumbar segments of the spinal column
angle	knee angle	angle between greater trochanter, lateral epicondyle, and lateral malleolus [24]

### 2.5. Statistical Analysis

The relationship between the change of skeletal angles and the actual instantaneous values of gravity or jerk was statistically analyzed. Both parameters of gravity and jerk were synchronized with the moment of each X-ray photo frame. Correlations were analyzed by the Pearson correlation coefficient with Fisher’s *r* to *z* transformation. The rates of gravity change were categorized by every 0.1 *j*. The statistical significance of the measurements was analyzed by the Kruskal–Wallis test, followed by the Mann–Whitney U-test. 

## 3. Results and Discussion

### 3.1. Mouse Skeletal Angles in Parabolic Flight

**Figure 4:**
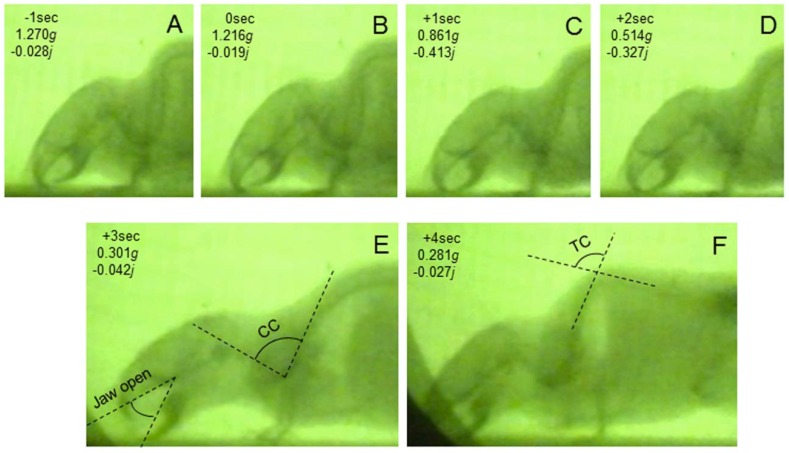
X-ray image of a mouse around the “dive” phase in the 0.3 *g*-targeting parabolic flight. In each frame, the actual time after the “dive” phase started was indicated at the top, the actual gravity level (instantaneous value) was shown in the middle, and the actual jerk value (optimized instantaneous value) was indicated at the bottom.

Upon entering into the “dive” phase of parabolic flight, all of the mice showed a typical pattern of postural change, initial extension and later flexion, regardless of the target-gravity level (Figure 4). Some mice showed the jaw-opening reflex during the “dive” phase of parabolic flights targeting <0.3 *g*. In the 0.3 *g*-targeting parabolic flight, a mouse showed the initial flexion (Figure 4A–D), followed by extension with the appearance of a jaw-opening reflex (Figure 4E) and then bending of the thoracic curvature with closing of the mouth (Figure 4F).

**Figure 5:**
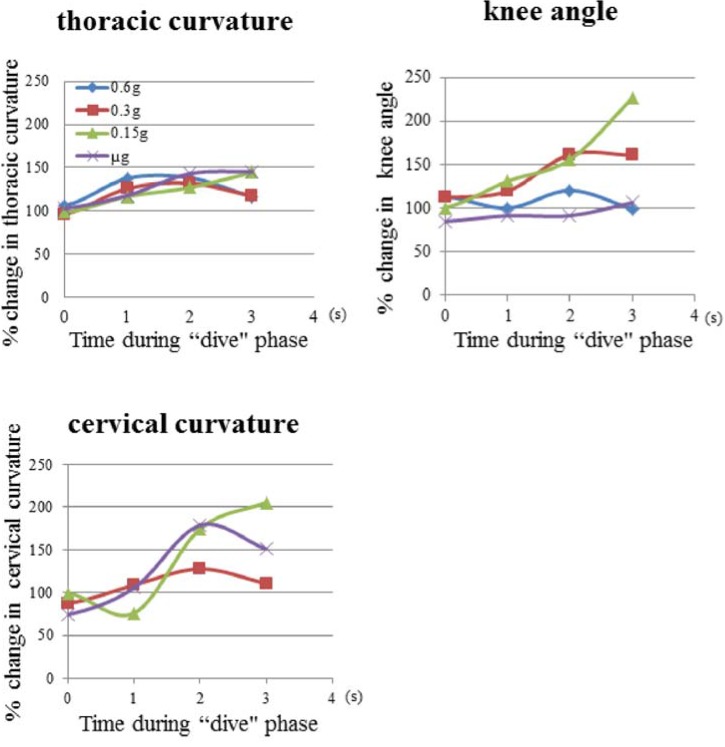
Changes in skeletal angles of thoracic curvature, knee angle and cervical curvature, during the “dive” phase of parabolic flights. The value shows the mean in the thoracic curvature (*n* = 6), knee angle (*n* = 3) and cervical curvature (*n* = 3). *n* = number of mice.

The gross outline of thoracic curvature (TC), cervical curvature (CC) and knee angle (KA) showed mouse differential responses to gravitational alteration in the “dive” phase of the parabolic flight (Figure 5)*.* KA and CC increased sharply for the first two seconds in the “dive” phase of the 0.15 *g*-targeting flight and stretched 200% at the time of 3.0 s. KA also increased in response to the 0.3 *g*-targeting flight, but had no response in the flights targeting 0.6 or μ *g*. CC stretching increased until 2.0 s in the “dive” phase of the 0.3 *g*-targeting flight, but not so much as in the flights targeting 0.15 or μ *g*. On the other hand, TC stretching appeared within a smaller range between 100% and 130% for all the parabolic flights. TC extension peaked at the times of 1.0 and 2.0 s and deteriorated at 3.0 s during the parabolic flights targeting 0.6 and 0.3 *g*. TC response in the “dive” phase of the flights targeting 0.15 and μ *g* was not as large as in targeting 0.6 and 0.3 *g*. TC, CC and KA showed a differential response to gravitational alteration in the “dive” phase of the parabolic flights*.*

### 3.2. Thoracic Stretching

**Figure 6:**
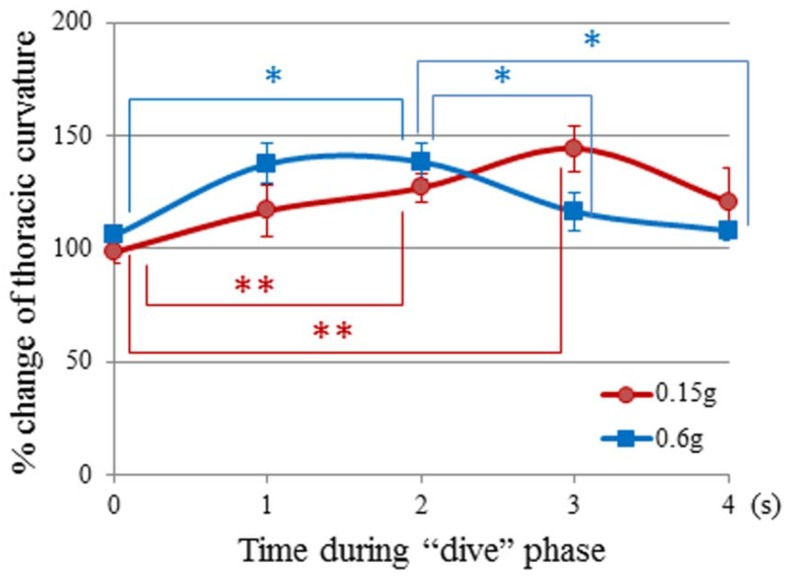
Time history of relative change in thoracic curvature in the “dive” phase of parabolic flight. All values are displayed as the mean ± SE (*n* = 6). Asterisks show statistically significant differences against the ‘starting-point control’ in the Kruskal–Wallis test, followed by the Mann–Whitney U-test (* *p* < 0.05, ** *p* < 0.01); *n* = number of mice.

As for the TC stretching, the higher responsiveness at mild low-gravity and lower responsiveness at severe low-gravity cannot be explained by the gravity alone. Another factor besides gravity was suspected to be involved in the mouse adaptation to gravitational alteration. Thus, the change of TC angle in the “dive” phase was statistically analyzed by focusing on gravity and jerk (Table 2, Figure 6).

**Table 2:** The angles of thoracic curvature at the time (seconds) in the “dive” phase of each parabolic flight. All values are displayed as the mean ± SE (*n* = 6).

Time (s) in the “dive” phase	0.6 *g*-flight	0.3 *g*-flight	0.15 *g*-flight	μ *g*-flight
−1.0	94.4º ± 5.2	82.9º ± 6.2	93.3º ± 6.3	89.9º ± 6.5
0.0	94.7º ± 4.6	86.1º ± 4.4	88.1º ± 4.7	91.9º ± 4.3
1.0	122.9º ± 8.8	112.6º ± 8.4	104.4º ± 11.6	105.4º ± 14.3
2.0	123.7º ± 8.7	118.0º ± 7.3	113.5º ± 6.4	127.8º ± 8.7
3.0	104.0º ± 10.4	105.1º ± 14.7	129.0º ± 10.2	130.1º ± 7.7
4.0	96.6º ± 3.5	99.81º ± 3.8	107.9º ± 10.7	96.1º ± 7.3

### 3.3. Time History of Gravity and Jerk

**Figure 7:**
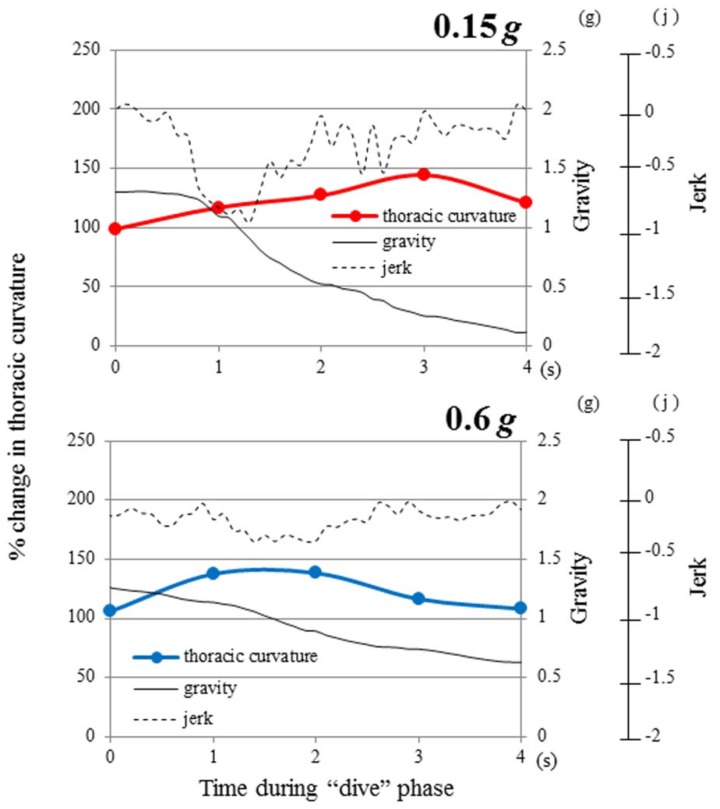
Time history of gravity and jerk in the “dive” phase of parabolic flight. The relative change of thoracic curvature in the “dive” phase is shown for the 0.6 *g*-targeting flight (blue line) and the 0.15 *g*-targeting flight (red line). The actual gravity level was calculated from the gravity values that were measured by gravity accelerometers in the x-, y-, z-axes at the sampling rate of 10 Hz. The jerk was obtained by averaging a total of 10 instantaneous values of the gravity acceleration rate in the range of 0.5 s before and after the exact moment that corresponded to the X-ray photo frame taken at 25 Hz.

**Figure 8:**
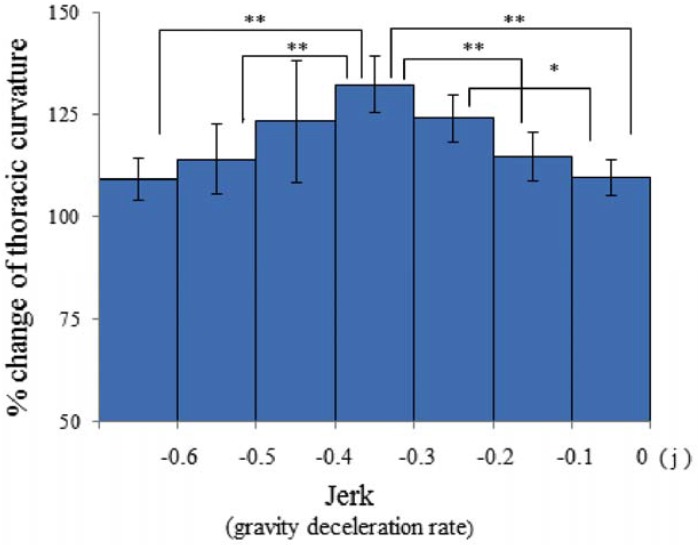
The effects of the negative jerk on thoracic-curvature extension in mice. All values are displayed as the mean ± SE (*n* = 6). Asterisks show statistically significant differences against the “0 jerk” control in the Kruskal–Wallis test, followed by the Mann–Whitney U-test (* *p* < 0.05, ** *p* < 0.01). *n* = number of mice.

The TC extension in mice was induced by parabolic flights targeting 0.6 *g* (*p* = 0.02), and 0.15 *g* (*p* = 0.006). On the 0.6 *g*-targeting flight, TC extension could be seen from the starting point of the “dive” phase (at 0.0 s), reached 130% at the timeframe of 1.0 s in the “dive” phase and sustained for the following 1.0 s (Table 2, Figure 6). The gravity level was 1.10 *g* and 0.80 g at the timeframes of 1.0 s and 2.0 s, respectively. As these gravity levels were not sufficient to induce the mouse response, what was the responsible factor for triggering TC stretching to 130% at the timeframes of 1.0 s and 2.0 s? The probable answer would be “−0.2 *j*” and “−0.3 *j*” at these points. In fact, the force by “−0.2 *j*” and “−0.3 *j*” significantly increased the TC stretching to 125%–130% (*p* < 0.05, *p* < 0.01, Figure 8). In the next one second towards the timeframe of 3.0 s, the TC stretching decreased significantly (*p* < 0.05). The gravity level was 0.7 *g*, due to a significant decrease (*p* < 0.01) of the gravity-deceleration rate from −0.3 *j* to −0.1 *j* (Figure 8). This difference of jerk resulted in a smaller angle of TC stretching at 3.0 s than at 2.0 s in the “dive” phase of the 0.6 *g*-targeting flight. At the timeframe of 4.0 s after entering the “dive” phase, the TC stretching diminished back to the “pre-dive” control level (Figure 7). The gravity level (0.6 *g*) or the jerk level (−0.1 *j*) was not sufficient to induce TC stretching at the timeframe of 4.0 s. 

**Figure 9:**
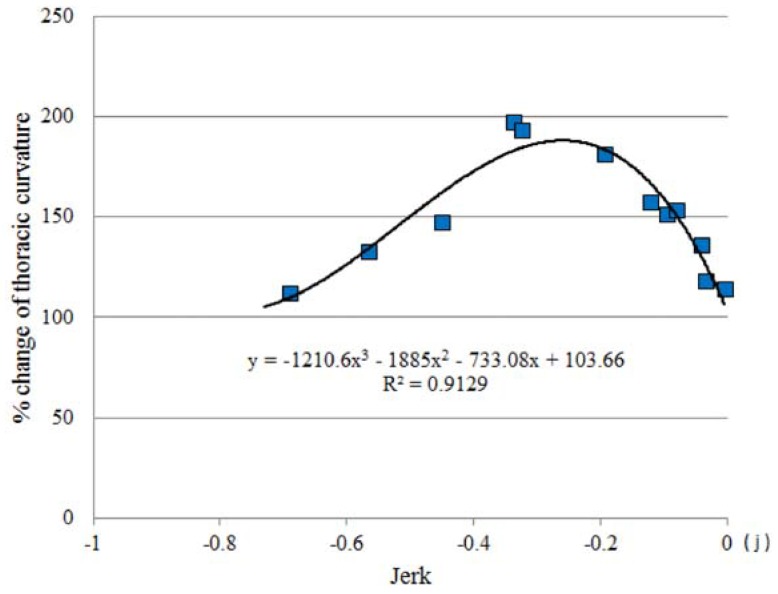
The limitation of mouse thoracic-curvature extension by negative jerk. The maximum response point of thoracic-curvature extension to each jerk level was displayed. Data form a curvilinear regression line, indicating a biphasic relationship between jerk and the limit of skeletal stretching.

On the 0.15 *g*-targeting flight, TC was extending slowly, but significantly, to 120% at the time of 1.0 s in the “dive” phase (*p* < 0.05, Figure 6). The gravity level at this point was 1.10 *g*, which appeared not to be strong. On the other hand, although the actual jerk level was −0.8 *j* and four-fold stronger than the jerk level (−0.2 *j*) that induced 130% of TC stretching in the 0.6 *g*-targeting flight, it did not show a significant effect on the TC stretching in the 0.6 *g*-targeting flight. Mouse TC cannot respond appropriately to such a strong gravity deceleration (Figure 8 and Figure 9). Toward the time of 2.0 s and 3.0 s during the “dive” phase of the 0.15 *g*-targeting flight, the TC increased to 130% and 145% (*p* < 0.01, Figure 6). At the timeframe of 2.0 s in the “dive” phase, the gravity level was 0.5 *g*, which was not sufficient to induce the response. However, the gravity-deceleration rate was approximately −0.3 *j*, which was in the appropriate range for inducing the maximum TC extension (Figure 9) and induced the largest TC extension eventually. In the next one second toward the timeframe of 4.0 s, the gravity level dropped sharply to 0.15 *g*, but TC stretching diminished to the starting point level. This may be due to the weak gravity-deceleration rate of −0.1 *j,* since −0.1 *j* was not sufficient to induce TC stretching (Figure 8 and Figure 9). At the timeframe of 4.0 s, the flight already was out of the “dive” phase and reached the target-gravity level of 0.15 *g*. The TC stretching in mice responded significantly to the gravity change in the initial phase of the 0.15*g*-targeting flight, before reaching the target gravity of 0.15 *g.*

### 3.4. Jerk and Thoracic Stretching

Mouse skeletal stretching by partial-gravity stimulation was correlated with gravity and jerk, *i.e.*, the gravity deceleration rate. The magnitude of the TC extension was regulated by jerk in a biphasic manner (*p* < 0.01, Figure 8). However, the maximum stretching was limited to 140%, due to the possible suppressive effects of extreme gravity deceleration (Figure 9). Mouse postural response to low gravity may undergo differential regulation by gravity and jerk. The most appropriate jerk for inducing the largest TC extension was approximately −0.3~−0.4 *j*, while weaker jerk (−0.1~0 *j*) or stronger jerk (~−0.6 *j*) did not show any impact (Figure 8). 

### 3.5. Regulation of Mouse Posture by Gravity and Jerk

The response pattern of the skeletal angles seemed to be partially influenced by the gravity level of the parabolic flight. However, the gravity alone cannot explain some results, such as the higher responsiveness in mild low-gravity and the lower responsiveness in severe low-gravities. In this context, we further examined this in detail, particularly by focusing on both the actual gravity level at 10 Hz and the actual jerk at 10 Hz, as each instantaneous value on the moment of the X-ray photo frame that was taken at 25 Hz. 

**Figure 10:**
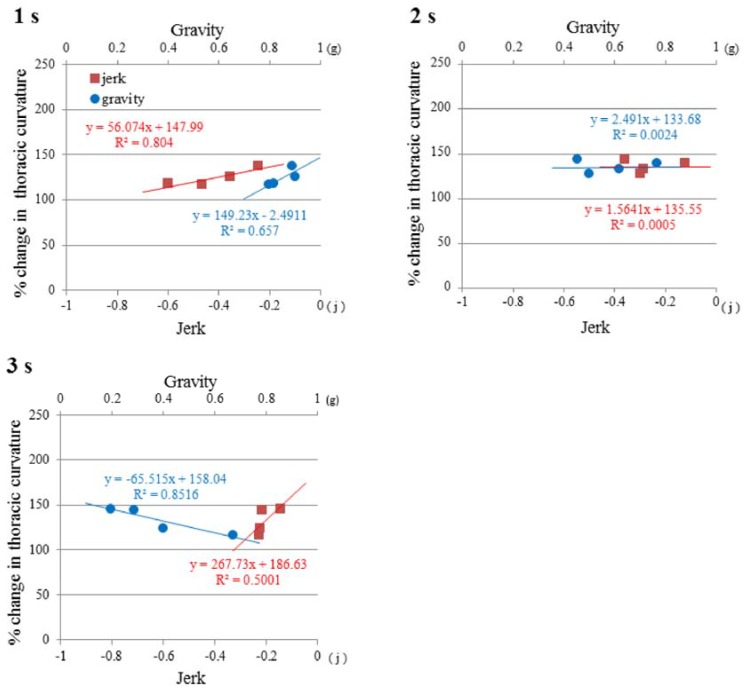
Correlations between thoracic-curvature extension and gravity or jerk. The graphs show the correlations at the timing of one (1 s), two (2 s) and three (3 s) seconds after starting the “dive” phase. The actual gravity level was calculated from the gravity values that were measured by gravity accelerometers in the x-, y-, z-axes at the sampling rate of 10 Hz. The jerk was obtained by calculating the average of 10 instantaneous values of the gravity acceleration rate in the range of 0.5 s before and after the exact moment that corresponded to the analyzed X-ray photo frame taken at 25 Hz.

As compared to the CC and KA, twice as many measurements were conducted on TC. We made further analyses on the correlation between TC and gravity or jerk, *i.e.*, the gravity acceleration rate in the “dive” phase of parabolic flights (Figure 10). At 1.0 s after the starting point of the “dive” phase, TC showed a positive linear correlation with gravity (a correlation coefficient R = 0.811), and with jerk (R = 0.897). The more gravity or jerk decreased in the range of *g* < 1.0 and *j* < 0.0, the more the thoracic curvature was reduced. The mouse TC was extended up to 150% by the mild low-gravity condition (0.9 *g*) and the mild jerk (−0.2 *j*). However, the TC-stretching effects of low-gravity and gravity-deceleration were getting smaller at lower gravities than 0.8 *g* or a bigger jerk of −0.6~−0.3 *j*. 

At 2.0 s after entering the “dive” phase, the thoracic curvature did not show any relationships with gravity (R = 0.002) or the gravity acceleration rate (R = 0.000). Around this moment, the TC extension was sustained at 140% regardless of alterations in gravity of 0.5~0.8 *g* or the gravity acceleration rate of –0.4~–0.1 *j*. At 3.0 s after entering the “dive” phase, the TC resumed a positive relationship (R = 0.707) with a gravity-acceleration rate of –0.22~–0.15 *j*. By contrast, TC showed a negative linear correlation (R = –0.928) with gravity. TC was extended by 150% at a gravity of 0.2 *g*. At 3.0 s after entering the “dive” phase, the flight was about to get out of the “dive” phase and move into the “target-gravity” phase. The gravity-acceleration rate was not volatile at the moment that TC was stretched and sustained as 140% regardless of alterations in gravity (0.55~0.8 *g*) or the gravity deceleration rate (0.1~0.4 *j*).

As a summary, the mouse TC extension in the “dive” phase of parabolic flight showed at least three patterns of relationships with gravity and jerk (Figure 10). Mouse postural response to low gravities may undergo differential regulation by gravity and jerk.

Previous studies have suggested postural adjustments to occur in bigger rodents (rats and hamsters) and other experimental animals in relation to gravity manipulation [17,22]. However, the effects of gravity on animal posture were observed only under relatively severe conditions, such as partial-gravity levels ≤0.15 *g* [17]. In the present study, significant stretching of the thoracic curvature was observed even at slow rates of gravity deceleration, which were associated with parabolic dives targeting the partial-gravity levels of 0.60 and 0.30 *g*. This means that postural adjustments occurred during the initial phase of gravity change; therefore, it is possible that postural adjustments may be more readily elicited in mice than in other larger animals in response to gravity deceleration. 

Mice had a tendency for coordinated extensor reflexes throughout the body when challenged with gravity deceleration. Earlier studies indicate that animals exposed to µ *g* are capable of maintaining normal body orientation, as long as a foothold is available [22]. In this situation, gravity sensing is provided by otolith receptors in the vestibular organ [23], by proprioceptive receptors present in the skin, muscles and joints [12], by visual photoreceptors [25] and possibly complemented by cardiovascular mechanoreceptors, as well [26]. It is likely that the postural adjustments observed here resulted from reflexes triggered by gravity-sensing receptors. Further studies will clarify the precise mechanism underlying animal adaptation to various gravitational conditions in space.

### 3.6. Limitations of This Study

Unfortunately, some technical limitations related to a parabolic flight study may have potentially interfered with our results. The first limitation was the number of animals enrolled in this study and the total number of observations recorded; still, the measured responses were consistent enough to yield statistically significant differences. In a few instances, the animals moved away from the active surface of the image intensifier, so that the head or the hindlimbs were not visible; some measurements were missed for this reason. In the present study, a precise (millisecond-based) synchronization of a G-accelerometer and the high-definition X-ray video frames enabled detailed analyses of mouse responses to exactly instantaneous levels of gravity and jerk. It is important to point out that the results obtained in parabolic flights are the product of short-lived transient changes in gravity; such experiments provide us with clues to the motor organization and reflex responses to gravity manipulations (more specifically, the transition into partial gravity) that may not necessarily correspond to the mechanisms involved in long-term adaptation to partial-gravity environments. 

## 4. Conclusions

Dramatic changes in animal posture occurred during the first few seconds in the “dive” phase of the parabolic flight when the gravity level was rapidly decreasing. The initial changes in body posture, such as the thoracic-curvature extension, were correlated with gravity and jerk, *i.e.*, the rate of gravity deceleration. The mouse postural response to low gravity may undergo differential regulation by gravity and jerk. Data suggest that postural reflexes are triggered readily at the onset of gravity change, and precede the disorientation phenomenon observed in animals in space. 
